# The Child Health PSO at 10 Years: An Emerging Learning Network

**DOI:** 10.1097/pq9.0000000000000449

**Published:** 2021-07-28

**Authors:** Fiona H. Levy, Katherine A. Conrad, Carol Kemper, Michaeleen Green

**Affiliations:** From the * Sala Institute for Child and Family Centered Care; † Department of Pediatrics, Hassenfeld Children’s Hospital at NYU Langone, New York, N.Y.; ‡ Delivery System Transformation, Children’s Hospital Association, Lenexa, Kans.; § Service and Performance Excellence, Children’s Mercy Kansas City, Kansas City, Mo.; ¶ Performance Measurement, Ann & Robert H. Lurie Children’s Hospital of Chicago, Chicago, Ill.

## Abstract

**Introduction:**

The 2005 Patient Safety and Quality Improvement Act, actualized as a Learning Network (LN), has enabled the Child Health Patient Safety Organization (PSO) to play a vital and novel role in improving the quality and safety of care. This article describes the Child Health PSO and proposes PSOs as a new construct for LNs.

**Methods:**

A PSOs ability to affect patient care depends on member organizations’ integration of PSO output into their individual Learning Healthcare Systems. Therefore, the Child Health PSO developed tenets of an LN to improve member engagement in PSO outputs.

**Results:**

All Child Health PSO members participate in case-based learning, requiring ongoing and robust participation by all members. The engagement has been strong, with 86% of children’s hospitals achieving a case learning activity metric and 60% of children’s hospitals submitting cases. From this LNs perspective, 53% of children’s hospitals are considered highly engaged.

**Conclusions:**

In the last 10 years, the Child Health PSO has evolved as a viable LN and, to sustain this, has set a target of 100% of participating children’s hospitals being highly engaged. The previously inconceivable notion of sharing information to improve patient safety among hospitals is now an expected result of the formation of trusting relationships under a federally certified PSO. According to participants, collaboration is an essential element that empowers individual children’s hospitals to eliminate preventable harm.

## INTRODUCTION

A Learning Healthcare System (LHS), as described by the Institute of medicine, is created from the application of timely evidence-based clinical decisions to drive individualized care better.^1^ In its 2013 report *Best Care at Lower Cost*, the Institute of Medicine codified the importance of shared knowledge and continuous learning to improve healthcare outcomes, quality, and equity and to lower costs. This approach is characterized by the use of real-time knowledge supporting clinical decision-making, the engagement and empowerment of patients, the transparency of data, and a leadership-instilled culture of learning.^2^ More recently, Learning Networks (LNs), which consist of multiple participating organizations working within a network architecture, have emerged to support the activities and outcomes of participant LHSs. These LNs are characterized by (1) the alignment of participants around a common goal; (2) policies, processes, and resources to enable multiactor collaboration; and (3) information sharing.^3–5^

The Child Health Patient Safety Organization (PSO) is a nonprofit subsidiary of the National Association of Children’s Hospitals, commonly referred to as the Children’s Hospital Association (CHA). On May 13, 2009, federal certification under the Patient Safety and Quality Improvement Act enabled a pioneering group of children’s hospitals to adopt a systematic approach to the detection and mitigation of harm risk to pediatric patients, which led to the formation of the Child Health PSO. This PSO is built on a history of collaboration among children’s hospitals to achieve better, safer, and more reliable patient care.^6–14^ It is designed to capture and learn from voluntarily reported infrequent preventable serious harm events occurring in children with diverse medical conditions. Preventable serious harm events are consistent with the Joint Commission’s Sentinel Event Policy description of events that result in permanent harm or serious temporary harm that is not related to the patient’s illness or underlying condition.^15^ This PSO is unique compared with other pediatric LNs, which frequently focus on patient-level clinical data to drive disease-specific collaborative improvements in care for single conditions and other targeted groups of hospital-acquired illnesses and injuries.^8–14^

This article describes the formation, growth, and maturation of the Child Health PSO. We communicate its evolution into a unique LN construct, discuss how organizers overcame hurdles unique to PSOs, and report on progress.

## METHODS

The methods employed in this investigation focused on LNs’ identifying characteristics: alignment around a common goal, multiactor collaboration, and information sharing. The following sections examine each of these in turn.

### Alignment of Participants around a Common Goal

For the Child Health PSO to affect patient safety and quality of care in children’s hospitals, participating organizations had to be willing and able to adopt a common goal of eliminating preventable serious harm, commit to submit safety event case detail to the PSO, and integrate PSO output into their delivery of care. Progress toward this goal began in 2011 with an in-person meeting of PSO members and with the application of federal protections allowing event learning from reported events. Although infrequent in any single organization, these events posed significant potential risks to pediatric patients across all member organizations. Participants endorsed the reporting and sharing of safety event data to better understand and address a broad range of pediatric safety risks independent of patient age, illness, or hospital-acquired condition. Children’s hospitals began to recognize otherwise unforeseen risks based upon learning that occurs within the structures that support multiactor collaboration described in section “Multiactor Collaboration and Information Sharing.” They recognized a unique opportunity to transition the PSO from a large pediatric data repository to a partnership among participating children’s hospital safety leaders that would support the elimination of preventable, serious harm to pediatric patients.

**Fig. 1.**
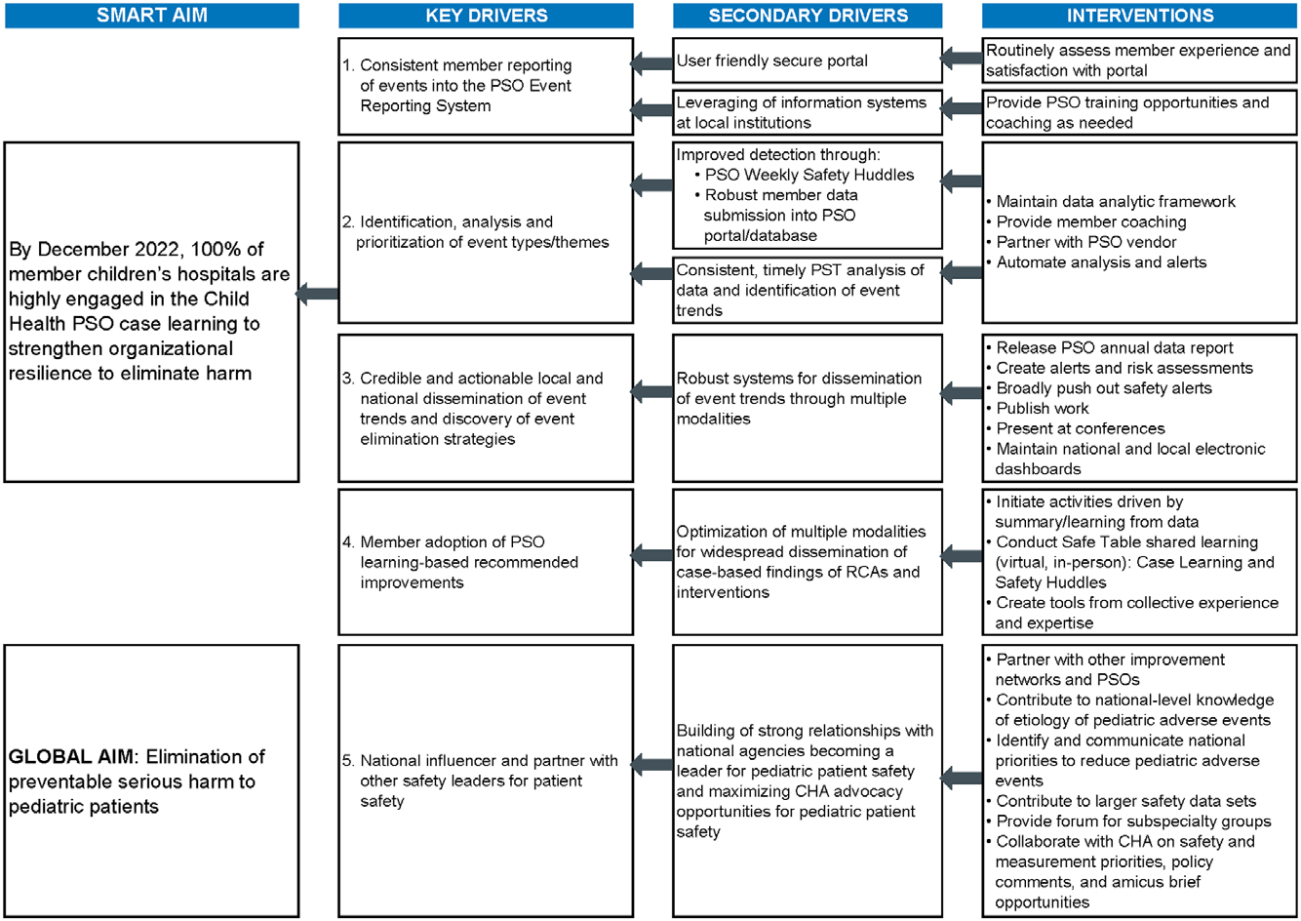
Depicts the child health PSO key driver diagram, which supports member engagement to achieve the global aim and describes PSO efforts in the key, primary, and secondary drivers, each building off the previous driver, from right to left. 1. An easy-to-use system and minimum data collection emphasize engagement on actionable shared learning, thereby demonstrating value from voluntarily reported data. 2. Experiences leveraged of those pediatric safety experts who could volunteer consistently for analysis and theme detection. 3. Although confidentiality of identifiable events must be preserved, national awareness of network findings is important to benefit children cared for in all settings. 4. Learning provides easily replicable, risk-mitigating strategies shared through regularly scheduled learning activities. 5. Duplication of efforts and resources to externally improve safety is minimized and other external safety efforts strengthened when goals align. RCA, root cause analysis.

The formalization of the voluntary PSO Patient Safety Team (PST), which followed, was another important step toward achieving a common goal. PST members are volunteer pediatric safety experts, all experienced in applying safety science when analyzing serious harm events occurring in children’s hospitals. The team created a key driver diagram (KDD)^16^ (Fig. 1) modified through multiple iterative reviews by external sources, including quality and safety leaders from the PSO participant organizations, members of the PSO Board of Directors, and the CHA Board Committee for Quality and Safety.

Throughout, the elimination of preventable serious harm to pediatric patients remained the global aim of the KDD designed to support a PSO LN. The specific and measurable, motivating, attainable, relevant, and trackable and time-bound (SMART)^17^ aim evolved to reflect a growing understanding of the PSOs limited positional authority over participants to reduce specific harm events and recognize that the primary value of the PSO depended on case reporting, participation, and engagement by member organizations. A review of the PSO KDD reveals the premise that reducing patient harm in a children’s hospital LHS can be achieved by optimizing participant engagement in the LN.

Recognizing that actionable change in the safety of care to children depends on a member’s case submission to the PSO and participation in additional case learning activities, investigators created a composite outcome metric, “engagement,” to establish an actionable and meaningful performance measure. The SMART Aim then became “100% engagement” of PSO member institutions by the end of 2022. To meet the composite engagement metric, a participating hospital is required to have reported 2 or more cases in 12 months and engaged in 2 of the 3 additional case learning activities: attended 80% of scheduled case learning events, attended the annual in-person meeting, and presented a case once every 3 years.

### Multiactor Collaboration and Information Sharing

The PST reviewed and analyzed submitted case narratives leveraging common cause-analysis methods. Submitted cases had undergone a “standardized root cause analysis process”^18^ within the reporting hospital. This standard process was adopted by most participating children’s hospitals trained through a separate organization, Children’s Hospitals’ Solutions for Patient Safety. Cases were then reviewed individually and collectively by PST members to maximize recognition of risk patterns and causal factors, such as lack of situational awareness, failure to communicate effectively, process failures, and errors in decision-making. Using qualitative systematic processes for consensus during event reviews, additional categories were contemplated that were not clarified using existing taxonomies.

The PSTs role and data analytics process positioned the PSO as an early warning system to detect potential areas of risk related to the care of children. The PST standardized multiactor collaboration on high-priority risk areas using three methods of learning and information exchange: safety alerts, safe tables, and safety tools, all designed to influence member adoption of best safety practices.

#### Safety Alerts

With the prevention of repeat safety events in mind, the PSO created safety alerts in 2012 to support member organizations’ ability to recognize the potential for, and then prevent, harm events at other participating children’s hospitals. The alerts have provided information that allows organizations to conduct proactive self-assessments and implement mitigation strategies to prevent repeat serious safety events. To support the creation of individual alerts, we engaged individuals from member institutions with specific expertise in human factors science, cognitive bias, and diagnostic error in the development process. We also included clinical experts aligning with areas of risk identified, such as medication safety, care management, and surgical safety.

#### Safe Tables

“Safe Table” is a term coined by PSOs to denote their federally privileged and confidential convening activities. Protected safe tables are open only to PSO participants, so that they can learn from safety events either in person or virtually. Additional administrative and technical procedures are implemented to minimize risk of impermissible disclosures and comply with the regulation to preserve privilege and confidentiality protections of these activities. Safe table discussions are augmented through a virtual platform that supports member organizations sharing resources and asking questions of peer children’s hospitals.

The PSO operates 2 primary Safe Table activities:

*Case Learning.* Case learning uses 1 or 2 cases to highlight specific areas of risk, such as insufficient coordination of care, failures associated with hospital discharge and home medications, or procedural mishaps/retained foreign objects. Case learning began in 2011. In 2013, it was aligned to support children’s hospitals’ focus on high-reliability organization principles from their participation in Solutions for Patient Safety and reporting of the serious safety event rate to track outcomes.^10,19^

*Safety Huddles.* PSO safety huddles are a variation on the daily safety brief held by many participating hospitals and a novel example of a safe table.^20^ Designed to broaden and hasten opportunities for shared risk identification, participants voluntarily ask for help or report a risk warning to other organizations. These early warnings have involved device- and medication-related harm or near misses, outbreak trends, various process failures, unanticipated communication breakdown, and safety challenges associated with COVID response. Safety huddles, designed as a multicenter early warning system, provide opportunities for early intervention and prevention of harm during care for children. From the 2014 startup through 9 months of implementation in 2020, the PSO has conducted over 341 weekly Safety Huddles, each running less than 15 minutes, generating over 1,823 early warning reports that have alerted children’s hospitals of potential risks to assess and mitigate.

#### Safety Tools—Proactive Risk Assessments

In 2017, the PST, in collaboration with other subject matter experts, began developing safety tools designed to support risk assessment and mitigation for specific infrequent but significant events at individual children’s hospitals. Individual hospitals had previously conducted their proactive risk assessments based on a safety alert; however, development within the PSO enabled access to a broader level of expertise for the risk assessment to be more robust. Most recently, a safety toolkit on diagnostic safety was published.^21^

## RESULTS

**Fig. 2.**
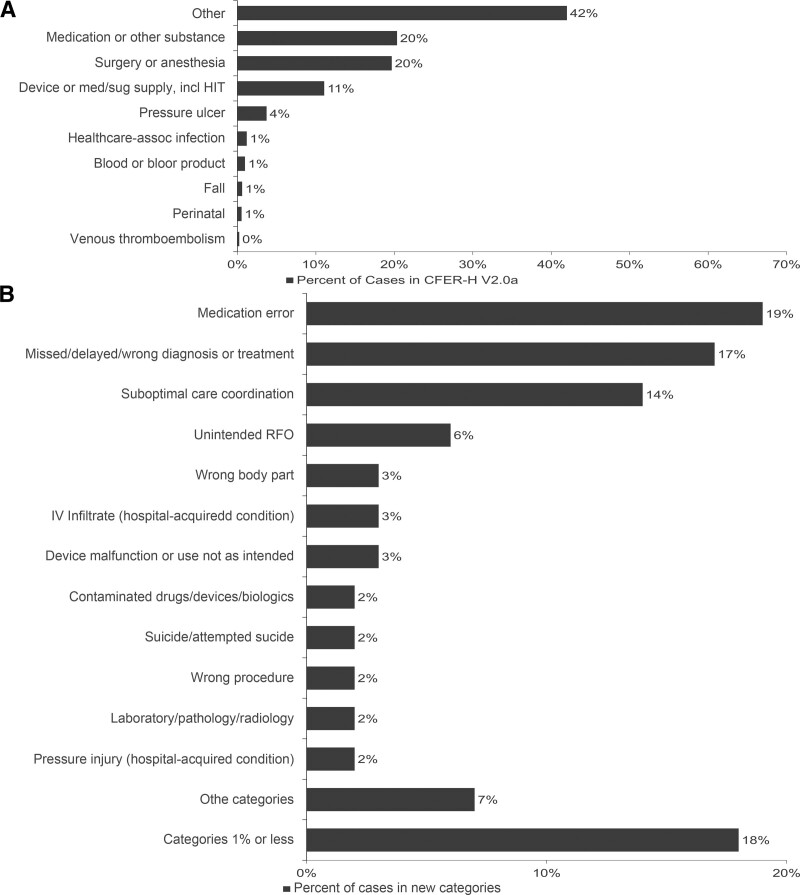
Categorization of 1,347 cases analyzed as of December 31, 2019. A, Results achieved using Common Formats for Event Reporting–Hospital Version 2.0 (CFER-H V2.0a) and reveals nearly half of reported cases fall within the "other" category. B, Results achieved using new categories developed by PST which allow assignment of all cases to specific themes enabling improved event-related learning.

The breadth and sometimes nonspecific nature of the safety event data, coupled with voluntary reporting, presented several challenges. One such challenge is illustrated in Figure 2. Among the 1,347 cases analyzed using AHRQ Common Formats for Event Reporting – Hospital Version 2.0 (CFER-H V2.0a),^22,23^ 562 cases fell in the nondescriptive category of “other,” resulting in 42% of our data camouflaging potential event-related learning. With improved delineation of the “other” category, we identified previously hidden case details and discovered essential themes, such as missed/delayed/wrong diagnosis and treatment, challenges of communication, suboptimal coordination of care, faulty decision-making, and diagnostic dilemmas. These themes resonated with the PST as often present in complex healthcare environments. As a result, we deployed these more specific categories to capture “other” cases and connect our data to learning and action relevant to children’s hospitals (Fig. 2A and B).

Before creating the engagement metric, PSO success was measured by reporting activity-based process measures, such as the percentage of members attending case learning events and huddles and case reporting for all active member hospitals. This initial way of monitoring PSO performance showed steady improvement in participation in attendance and reporting cases as a percentage of total members, which increased to 61 children’s hospitals in 2020.

**Fig. 3.**
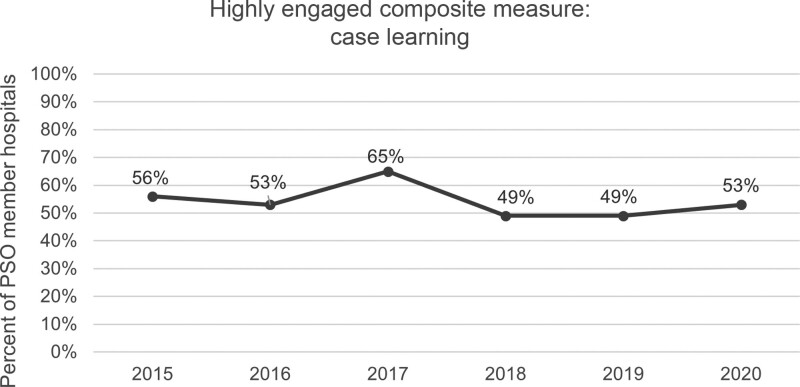
A new composite measure from an LN perspective improves understanding of engaged participation.

Our “highly engaged” composite measure provides a new view of PSO progress by factoring in the level of participation (or input) needed from each member to sustain the LN outputs to achieve the global aim from the participating organization’s perspective. Of 43 children’s hospitals participating in the PSO from 2015 to 2019, engagement steadily decreased from 56% in 2015 to 49% in 2018 and 2019, other than a peak of 65% in 2017. As of September 30, 2020, engagement has increased to 53% (Fig. 3).

**Fig. 4.**
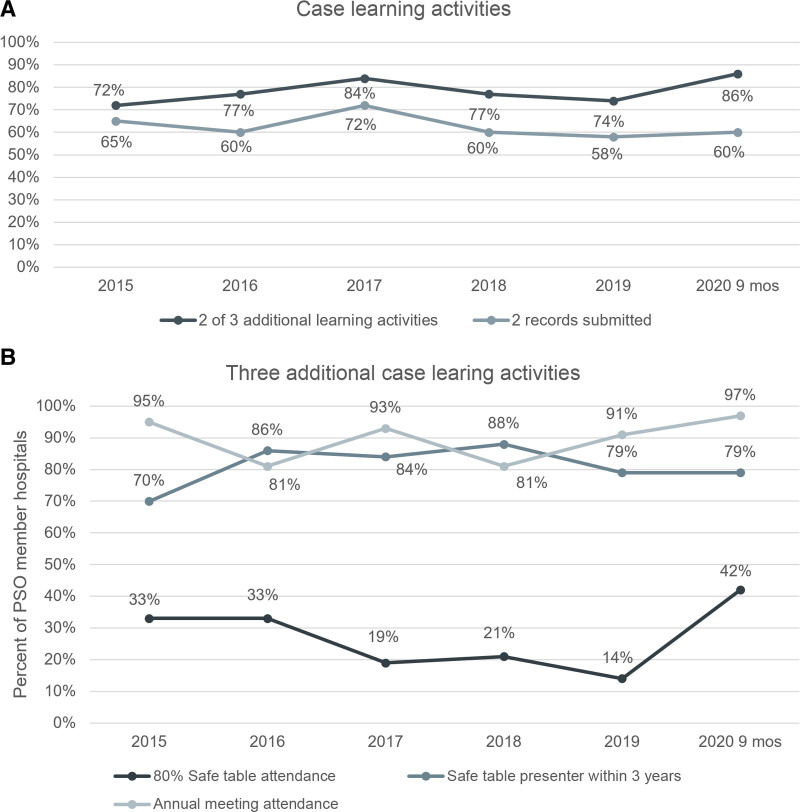
Analysis of performance across all components of the composite measure allows identification of targeted areas for improvement. A, Reports breakdown of composite engagement data into rates of case reporting and learning activity participation. B, Details engagement percentages for each of the three case learning activities.

Analysis of performance on the composite measure components reveals that member children’s hospitals reporting two or more case reports has increased from 58% in 2019 to 60% in 2020, following a peak of 72% in 2017. Achieving other case learning activities has increased from 74% of children’s hospitals in 2019 to 86% in 2020, following a peak of 84% in 2017. Annual meeting attendance by September 2020 reflected 100% of members registering for the October virtual annual meeting (97% ultimately attended, which did not impact measures overall). Member attendance in 80% of safe tables decreased significantly from 33% of children’s hospitals in 2015 and 2016 to a low of 17% in 2019, but this has improved to 42% as of September 30, 2020. Case presentation once every three years has remained steady at or above 79% of children’s hospitals since 2016, fulfilling a case learning teaching principle to which members contribute as part of the LN (Fig. 4A and B).

## DISCUSSION

We believe that member hospital engagement in the form of LN contributions and active learning from the LN is one of the necessary tools to drive safety improvement across children’s hospitals and, therefore, maximize value from the PSO. Although we set an ambitious SMART Aim of 100% of children’s hospitals being highly engaged in 2022 and targeting 60% in 2020, we believe improvement to 53% of children’s hospitals being highly engaged as of September 2020 is significant considering the children’s hospitals’ operational challenges resulting from the COVID-19 pandemic. The goal-based measurement allowed us to identify and begin to address areas needed for improvement within PSO processes. For example, modifications of our case learning designed to increase participation have included coaching presenters to share safety event case details and corrective actions that are replicable and actionable. Finally, in 2019, participant organizations were provided customized feedback on engagement performance to create shared accountability for the effectiveness of the PSO’s value as an LN supporting the LHS.^24^ The preliminary review of data shows engagement has increased over the last nine months.

Our partnership with 61 children’s hospitals was designed as a continuous PSO LN to strengthen participants’ organizational learning and support their efforts to detect and mitigate the risk of harm in patient care. It is commonly stated within the PSO that no child should experience a preventable safety event if learnings from another hospital’s event can prevent its occurrence. Yet, we recognize that the relatively infrequent occurrence of serious harm incidents, combined with an absence of condition-specific alignment, can challenge an organization’s efforts to identify and mitigate safety risks to their patients.^25^ When PSO-submitted event details are combined, collated, and analyzed, newly acquired knowledge of infrequent risks provides insight on priorities for improving safety. However, this requires sufficient, reliable data; participation in and contribution to learning opportunities, and overall network engagement (the LN). Also required is each organization’s willingness to prioritize and tailor their processes to implement specific safety interventions within their organizations (the LHS).

The PSO embraced many of the characteristics of disease-specific LNs, tailoring them as needed to realize its unique goal. We have achieved the 3 major components of an LN: (1) alignment of participants around a common goal; (2) policies, processes, and resources to enable multiactor collaboration; and (3) sharing of information to achieve the goal. By contemplating our lessons learned, we have demonstrated a new path for an action-oriented LN to support the elimination of serious pediatric harm. Future studies on member perspective of barriers to engagement, measuring children’s hospitals’ adoption of tools, and the impact of engagement to eliminate serious harm are all opportunities for broader understanding of the LN impact.

The last 10 years have seen the Child Health PSO evolve as a viable LN to support children’s hospitals’ LHSs. The sharing of information—historically considered inconceivable—is now an expectation embedded in the trust relationships formed within the PSO. The PSO allows for both mature and novel practices for information sharing and risk consciousness, resulting in a promising formula for collaboration. From the perspective of those actively participating, this collaboration is not just an option—it is now an essential element that further empowers individual children’s hospitals to eliminate preventable harm to pediatric patients. We believe that this is the real success of the PSO LN.

## DISCLOSURE

The authors have no financial interest to declare in relation to the content of this article.

## ACKNOWLEDGMENTS

The authors wish to thank past and present members of the Child Health PSO Patient Safety Team for their passionate commitment of time and effort, Barbara Weis for her persistent hard work and analytic skills in support of the PSO, and Angelo Giardino, MD, Ph.D., MPH, FAAP, Chief Medical Officer and Chair, Department of Pediatrics, Primary Children’s Hospital, and Troy Richardson, Biostatistician, Children’s Hospital Association, for their expert manuscript review.
